# Complete mitochondrial genome sequence and phylogenetic implications of *Chorosoma macilentum* (Heteroptera: Rhopalidae)

**DOI:** 10.1080/23802359.2019.1687029

**Published:** 2019-11-11

**Authors:** Wanqing Zhao, Dajun Liu, Zhizhong Gao, Jia Liu, Wenbo Yi, Hufang Zhang

**Affiliations:** Department of Biology, Xinzhou Teachers University, Xinzhou, China

**Keywords:** *Chorosoma macilentum*, Rhopalidae, next-generation sequencing, mitochondria genome, phylogeny

## Abstract

The complete mitogenome of *Chorosoma macilentum* Stål, 1858 was obtained using a next-generation sequencing approach, which is the first report in genus *Chorosoma*. The genome is 16,330 bp long with high A + T content. The phylogenetic analyses based on 13 protein-coding genes (PCGs) provide strong support for monophyly of each family within Pentatomomorpha. *Chorosoma Macilentum* was clustered together with other species within Rhopalidae, and grouped sibling species with *Aeschyntelus notatus*.

The genus *Chorosoma* Gurtis, 1830 belongs to Rhopalinae (Hemiptera: Heteroptera: Rhopalidae), threating the plants of Gramineous (Hsiao 1977). The distribution region of *Chorosoma macilentum* is rare, only distribute in Kazakhsta, Russia, Mongolia and China, whereas distribute in Inner Mongolia, Xinjiang, Gansu, and Shanxi province of China. Researches based on mitochondrial genome (mitogenome) have proven to be a useful tool for phylogenetic relationships, however, no mitogenome sequences of genus *Chorosoma* were available. Here, we obtained the complete mitochondrial genome of *C. macilentum* Stål, 1858 for the first time.

The specimen of *C. macilentum* was collected on 4 August 2016 from Jincheng city (112.013576 N, 35.335836E), Shanxi Province, and deposited in the Zoology laboratory of Xinzhou Teachers University, Shanxi, China (specimen accession No. LY1). The mitogenome was sequenced using the Illumina HiSeq2500 and annotated protein-coding genes by predicting open reading frames using the invertebrate mitochondrial code. Additionally, tRNA genes were identified based on their characteristic cloverleaf secondary structure using the MITOS web server (Bernt et al. 2013). Bayesian inference (BI) analysis was performed using MrBayes3.2 (Ronquist et al. 2012) under a GTR + G + I modelselected by jModeltest (Darriba et al. 2012).

The complete mitogenome is 16,330 bp in length (GenBank accession No. MN412595), containing one control region and 37 genes (including 13 PCGs, 22 tRNAs, and 2 rRNAs). The gene arrangement are identical to that of most Heteroptera species (Hua et al. 2008; Yuan et al. 2015), which is typical double-stranded circular moleculer with an asymmetric nucleotide composition (42.38% A, 13.26% C, 9.51% G, and 34.84% T). The AT-skew (0.098) for the whole mitogenome is slightly positive while GC-skew (−0.165) is negative, with an obvious A + T bias (77.23%). Among the 13 PCGs, only ATP8 *cox1* uses start codon TTG, the rest are encoded by the typical ATG (*nad2*, *atp6*, *cox3*, *nad4* and *cytb*), ATA (*nad3, nad5 and nad6*), ATC (*cox2* and *atp8*) and ATT (*nad4L and nad1*) start codons. Particularly, complete stop codon TAA/TAG was found in *atp8*, *nad4*, *nad4L* and *cytb*, while the rest were encoded by T. The length of 22 tRNAs ranged from 62 to 72 bp, and all have the typical cloverleaf structure except *trnS(AGN)* and *trnV*, which lack a dihydrouridine arm.

A Bayesian inference (BI) phylogenetic analysis with 28 Pentatomomorpha species and three Cimicomorpha species was conducted using 13 PCGs (Figure 1). The monophyly of each family were generally well supported on BI tree, and *C. macilentum* was clustered together with other species within Rhopalidae (PP = 1). Further study with more mitogenomes of Rhopalidae species will help in understanding phylogenetic status of this family.

**Figure 1.**
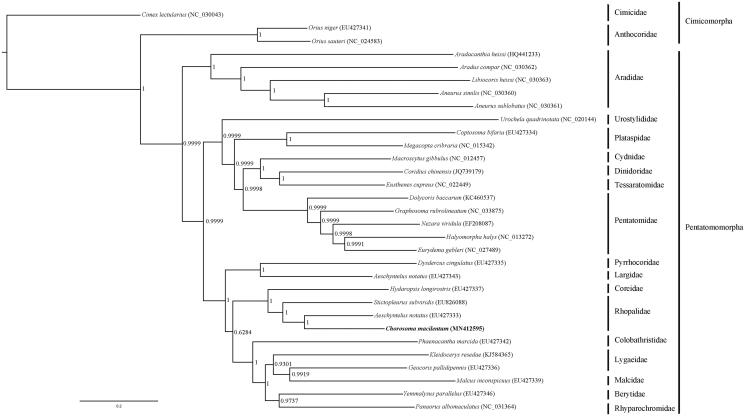
Phylogenetic relationship of *Chorosoma macilentum* within Pentatomomorpha inferred from 13 PCGs.
